# Decoding chromatin-associated RNA (caRNA) interaction networks: from genome-wide dynamic capture to epigenetic mechanisms in plants and animals

**DOI:** 10.1080/15592294.2026.2733375

**Published:** 2026-10-12

**Authors:** Can Liu, Wenze Li, Qi Lv, Rui Su

**Affiliations:** a College of Animal Science, Inner Mongolia Agricultural University, Hohhot, China; b Sino-Arabian Joint Laboratory of Sheep and Goat Germplasm Innovation, Hohhot, China; c Inner Mongolia Key Laboratory of Sheep & Goat Genetics Breeding and Reproduction, Hohhot, China

**Keywords:** Chromatin-associated RNA (caRNA), RNA-chromatin interaction, Genome-wide capture technology, epigenetic networks, 3D genome

## Abstract

Chromatin-associated RNAs (caRNAs) serve as central hubs for regulation within the cell nucleus and are involved in key biological processes such as transcriptional homeostasis, chromatin remodeling, and transgenerational inheritance. In recent years, the development of various genome-wide RNA-chromatin interaction capture technologies has made it possible to resolve the spatial interaction networks between caRNAs and chromatin with high precision. This paper systematically reviews the concepts, functions, and interaction patterns of caRNA, and provides a comprehensive overview of the principles, characteristics, technical biases, and applicable scenarios in the fields of targeted and non-targeted identification technologies. Building on this foundation, this paper conducts a comparative analysis of caRNA regulatory mechanisms in animals and plants across four functional dimensions: the organization of three-dimensional chromatin topology, RNA modification-mediated epigenetic regulatory switches, the transmission of environmental and transgenerational signals, and the pathological mechanisms associated with caRNA network dysregulation. Through this comparative analysis, this paper reveals the common characteristics of caRNA-mediated functional regulation in animals and plants, as well as lineage-specific adaptive strategies, and discusses the challenges and future directions in this field regarding single-cell resolution, AI-assisted data analysis, and translational applications.

## Introduction

According to the central dogma of classical molecular biology, genetic information is transmitted from DNA to RNA and then to proteins. However, less than 2% of the transcripts in eukaryotic genomes encode proteins [1]; the vast majority of the remaining transcripts are non-coding RNAs (ncRNAs). Most transcripts in eukaryotic genomes do not encode proteins but instead play potential roles as ncRNAs. Within the cell nucleus, there exists a class of RNA molecules that form stable physical interactions with chromatin, known as chromatin-associated RNA (caRNA). In recent years, caRNA has been shown to play a central role in processes such as transcriptional regulation, maintenance of chromatin higher-order structure, genomic stability, and transgenerational inheritance [2]. However, because chromatin is highly folded within the cell nucleus and interactions between RNA and chromatin are dynamic and transient, traditional biochemical methods struggle to capture these interactions at high resolution across the entire genome. Over the past decade, the rapid development of multi-omics technologies, based on proximity tagging, targeted enrichment, and deep sequencing has enabled researchers to systematically map the landscape of RNA-chromatin interactions.

With the rapid development of related research, systematic summaries have been conducted on technologies for mapping RNA-chromatin interaction maps and identifying caRNA [3], or the mechanism of action of caRNA in specific situations such as cancer [4]. However, there is currently a lack of systematic summaries on whether the regulatory mechanisms of caRNA exhibit universal or specific regulatory strategies between animals and plants. This review aims to fill this gap by adopting a comparative framework. It critically evaluates the conservation and differentiation of common mechanism dimensions such as R-loop formation, phase separation condensate formation, and m^6^A-dependent chromatin recruitment during evolution. This cross-organism perspective not only reveals the core mechanisms of caRNA-mediated regulation but also opens up space for future interdisciplinary translational research, applying the research results of plant epigenomics to crop improvement or utilizing animal cancer models to understand plant stress responses. This review aims to systematically summarize the identification technology innovations and cross-species regulatory mechanisms in the field of caRNA. Firstly, it elucidates the core concepts and spatial interaction modes of caRNA, comprehensively describes the current mainstream targeted and non-targeted identification technologies, and provides methodological comparisons and applicability guidelines. On this basis, this article comprehensively analyzes the pathological mechanisms and transgenerational inheritance of caRNA interaction networks in animal models, as well as the regulatory mechanisms of environmental adaptation and circadian rhythms in plant models. Finally, it discusses the current technical bottlenecks faced in this field and provides an outlook on future development directions.

## The concept and function of caRNA

### The concept and classification of caRNA

Previous studies have shown that more than 75% of the human genome is transcribed, but less than 1.5% of these regions encode proteins [1]. This suggests that the transcription process not only produces housekeeping RNAs that sustain vital functions, but that non-coding RNAs also possess regulatory potential. ncRNAs encompass many categories, such as snoRNAs, snRNAs, miRNAs, lncRNAs, and eRNAs. Notably, the cell nucleus contains a large number of RNA molecules that are transcribed but not processed before exiting the nucleus; these molecules stably and tightly bind to chromatin. Such molecules are collectively referred to as chromatin-associated RNA (caRNA) [2]. However, caRNA is not a single molecular subpopulation but rather a complex collection of transcripts of various biological types that play roles in chromatin maintenance and gene regulation. caRNA includes both mRNA and ncRNA; nascent pre-mRNA from the co-transcriptional stage accounts for more than 90% of this group, while the remaining approximately 10% consists of ncRNA, which also plays an important role [5].

Traditionally, pre-mRNA is considered an intermediate state in genetic information transmission, representing the unprocessed form of mature mRNA. However, GRID-seq data reveals that over 90% of RNA enriched on chromatin is pre-mRNA [2]. These caRNA derived from precursor mRNA constitute a diverse group, including intragenic antisense transcripts, promoter upstream transcripts (PROMTs), and partially spliced or unspliced precursors retained at the transcription site [5]. Although their typical role is as intermediates in protein synthesis, an emerging viewpoint suggests that specific precursor mRNA can also function as non-coding structural RNA, regulating local chromatin states [6].

Beyond mRNA-derived pre-mRNA, various types of non-coding caRNA also play significant roles in genomic interaction networks. This review focuses primarily on caRNAs of the ncRNA class; content related to pre-mRNA is addressed only in representative cases. Long non-coding RNA (lncRNA), with lengths exceeding 200 nt, is gradually being explored by researchers as the most functionally complex type of caRNA. Due to its long sequence serving as a highly modular molecular scaffold, it can physically link different epigenetic modification enzymes and precisely deliver them to specific genomic target sites. Small nuclear RNA (snRNA) and small nucleolar RNA (snoRNA) are two crucial types of non-coding RNA within the eukaryotic cell nucleus, playing irreplaceable roles in RNA post-transcriptional processing and modification. snRNA is typically enriched in the spliceosome of the cell nucleus, where it combines with various core proteins to form the small nuclear ribonucleoprotein complex, which is central to pre-mRNA splicing in eukaryotes. snRNA ensures the accuracy of mature mRNA sequence by base-pairing with the splice sites within the intron region of pre-mRNA, facilitating intron excision and exon splicing. snoRNA, on the other hand, is primarily localized in the nucleolar region of the cell nucleus, mainly guiding other RNA molecules for specific chemical modifications [7]; Enhancer RNAs (eRNAs) are produced by bidirectional transcription of active enhancers and range in length from approximately 50 to 2000 nucleotides. They serve as markers of active enhancers, mediating the recruitment of proteins such as BRD4 to H3K27ac-modified regions through three-dimensional chromatin looping between enhancers and target genes, thereby activating the transcription process [8,9]. Promoter antisense RNAs (paRNAs) regulate the chromatin state of the promoter region by stabilizing histone demethylases KDM4B and KDM4C [10].

### Interaction patterns between caRNA and chromatin

Accurately defining the molecular basis of caRNA-chromatin interactions and their spatial scale within the genome is a prerequisite for elucidating their biological functions. At the mechanistic level, the binding of caRNA to chromatin primarily involves the following three modes: direct RNA-DNA base pairing, in which caRNA forms R-loops or triple-helix structures with genomic DNA through sequence complementarity, thereby achieving sequence-specific physical binding; protein-mediated interaction, in which caRNA acts as a scaffold molecule to recruit chromatin-binding protein complexes, such as the polycomb repressive complex 2 (PRC2), the CCCTC-binding factor (CTCF), or Argonaute 4 (AGO4), indirectly anchoring it to specific chromatin regions; Spatial proximity effects within the nuclear compartment: Such associations are primarily captured using proximity-based techniques, and the detection results reflect the spatial proximity of RNA and DNA within the nucleus rather than direct physical contact.

At the genomic level, caRNAs can be classified into three categories based on the scope of their regulation of target sites: cis-acting, trans-acting, and cis-trans cooperative [2]. The following sections will systematically describe these three modes of action and simultaneously analyze the corresponding molecular binding mechanisms for each mode, thereby establishing a comprehensive framework for understanding caRNA function from both the perspectives of molecular interactions and the genomic scale. These three interaction modes and their corresponding molecular mechanisms are summarized in Figure 1.

**Figure 1.**
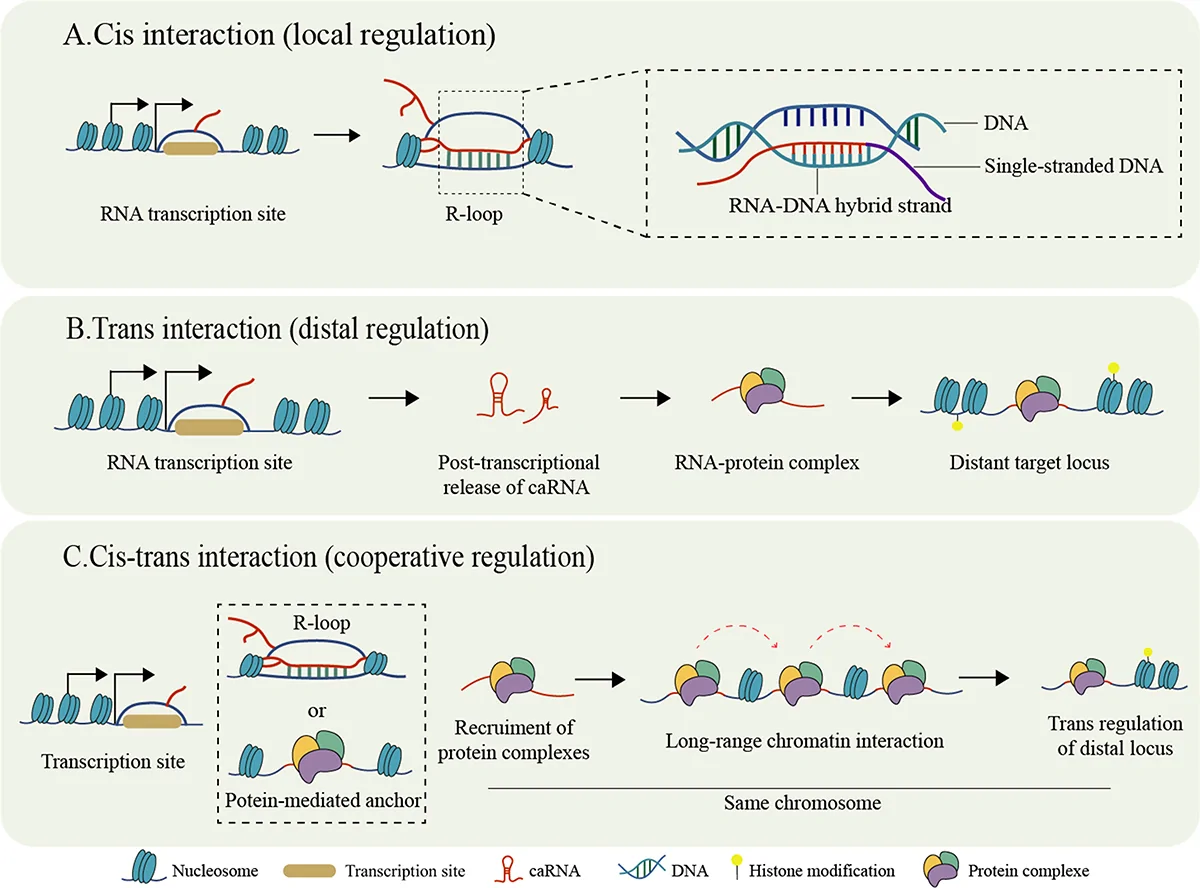
Different modes of RNA-chromatin interactions.

Description:(A) Cis-action: caRNA forms an R-loop via RNA–DNA base pairing and anchors itself near the transcription site; the figure shows a magnified view of the RNA–DNA hybrid strand within the R-loop and the displaced single-stranded DNA; (B) Trans action: After transcription, the caRNA is released and binds to a protein complex, thereby targeting a distant genomic site; (C) Cis-trans synergy: The caRNA is first cis-anchored near the transcription site via an R-loop or protein-mediated mechanism, and subsequently mediates trans regulation of a distant site on the same chromosome through long-range chromatin interactions mediated by the protein complex.

In cis-action, the molecular binding mechanism of caRNA typically involves direct RNA-DNA base pairing, whereby it anchors itself near the transcription site through the formation of an R-loop. caRNA exerts its regulatory effect near its transcription site by forming a three-stranded nucleic acid structure – known as an R-loop – composed of an RNA-DNA hybrid strand and a displaced single-stranded DNA strand [11]. To fully understand the regulatory mechanisms of caRNA, it is crucial to distinguish between physiological and pathological R-loops, which primarily depends on whether the cell maintains homeostasis. Physiological R-loops actively function in processes such as gene expression regulation, DNA replication initiation, and damage repair. However, if there are defects in the mechanisms of helicases or specific recognition proteins, or if RNA processing and export are impeded, leading to their abnormal accumulation, physiological R-loops become pathological. This results in the breakage of the displaced single-stranded DNA, defects in local chromatin remodeling, and genomic instability, thereby facilitating cancer transformation.

Unlike cis-action, the molecular binding mechanism of caRNA in trans-action typically relies on protein-mediated interactions, whereby caRNA acts as a scaffold to recruit chromatin modification complexes and direct them to distal genomic sites. Trans-action refers to the process in which protein-mediated caRNA, following transcription at a transcription site, interacts with other chromosomes or distal loci across the entire genome. This type of RNA transports chromatin-binding complexes and other factors to distal target regions, thereby regulating distal chromatin structure independently of the transcription site [2]; cis-trans synergy integrates these two molecular mechanisms. caRNA is first cis-anchored near the transcription site via an R-loop or protein-mediated mechanism, and subsequently diffuses over long distances along the same chromosome through the recruitment of protein complexes, thereby achieving trans-regulation. In summary, although the interactions between caRNA and chromatin exhibit diversity in molecular mechanisms and modes of action, their core function revolves around RNA serving as a dynamic molecular scaffold that, through sequence-specific or protein-mediated mechanisms, recruits and organizes chromatin modification complexes under specific spatiotemporal conditions, thereby forming a complex and dynamic RNA-chromatin interaction network.

## Techniques for identifying RNA-chromatin interactions

In recent years, advances and rapid developments in molecular biology-based identification techniques have enabled researchers to systematically identify and map caRNA-chromatin interaction networks. However, early experiments relied on biochemical purification methods, which had low sensitivity and were prone to capturing nonspecific false-positive binding fragments. In recent years, the continuous emergence of new technologies has made it possible to precisely identify RNA-chromatin interaction sites and elucidate their regulatory roles at both the single-locus and genome-wide levels. In caRNA research, identification techniques can be broadly categorized into two types based on their experimental principles: targeted and non-targeted. Targeted techniques use a specific RNA or DNA molecule as a starting point to track and capture regions that interact with it across the entire genome. Non-targeted techniques, on the other hand, do not involve a pre-binding step and capture all interacting regions across the entire genome.

It is worth noting that the captured RNA-chromatin interactions exhibit significant differences in their molecular nature, ranging from stable physical binding to spatial proximity. Specifically, hybridization-based capture strategies directly identify genomic binding sites for specific RNAs, whereas proximity-linking approaches primarily detect spatial colocalization of RNA and DNA within nuclear compartments. Importantly, the detection of chromatin binding through proximity-based detection methods does not necessarily imply direct or functional binding between RNA and chromatin. Furthermore, depending on the binding anchor, these techniques can be further classified into RNA-centric, DNA-centric, and protein-centric strategies.

### Targeted identification technology

RNA molecules play a crucial role in the transmission of genetic information, and their processing and maturation are typically associated with post-transcriptional gene regulation. There are also physical interactions between RNA and chromatin. Technologies for identifying caRNA can be further classified into two categories based on the type of anchor. One category targets specific RNA molecules, including the use of biotin-labeled antisense probes to hybridize with target RNAs and capture the chromatin-DNA complexes that interact with them, such as ChIRP-seq (Chromatin Isolation by RNA Purification) [12] and CHART-seq (Capture Hybridization Analysis of RNA Targets) [13]. The other category targets specific genomic regions, DNA-binding proteins, or histone modifications, including ChRD-PET (Chromatin-associated RNA-DNA interactions followed by Paired-End-Tag sequencing) [14], which is based on ChIP to target specific proteins or modifications, and TaDRIM-seq (Targeted DNA-associated RNA and RNA-RNA Interaction Mapping by sequencing) [15].

#### RNA-centered strategies

ChIRP-seq works by using biotin-labeled antisense oligonucleotide probes to bind to target RNA, capture DNA fragments that interact with it, and then perform high-throughput sequencing to identify the genomic regions to which the RNA is bound. This technique was validated in a fruit fly model using the lncRNA roX2 [12]. The targeted interactions of the lncRNA HOTAIR were analyzed in human fibroblast cell lines, revealing that HOTAIR physically colocalizes with epigenetic modification complexes such as PRC2 and LSD1 across the genome [16].

CHART-seq incorporates RNase H sensitivity assays into probe design and optimizes them to precisely locate highly accessible regions on target RNAs, thereby reducing probe usage while minimizing off-target effects and enhancing capture specificity [13]. Using this technology, the whole-genome binding map of Xist in mice was resolved at extremely high resolution, revealing that Xist first distributes to gene-enriched regions and then finely covers the entire X chromosome, thereby elucidating the dynamic process of lncRNA-mediated epigenetic silencing [17]. ChIRP-MS and CHART-MS combine RNA-targeted capture with mass spectrometry to simultaneously identify the protein networks bound to target RNAs [18,19]. HiChIRP further combines ChIRP with Hi-C to resolve the three-dimensional chromatin interaction networks mediated by specific target RNAs, using them as anchors; it exhibits extremely high sensitivity, capable of detecting chromatin interactions mediated by RNAs present at as few as approximately 10 copies per cell. It has also been demonstrated that specific lncRNAs can directly mediate the formation of chromatin loops [20].

Both techniques rely on the efficiency of probe-RNA hybridization and are therefore sensitive to the secondary structure and accessibility of the target RNA. CHART-seq, through RNase H-guided probe design, exhibits superior capture efficiency for highly structured RNA compared to ChIRP-seq. Neither method can distinguish between direct RNA-DNA binding and protein-mediated bridging, and both may capture non-specific spatial proximity interactions within nuclear compartments. Furthermore, probe design may introduce sequence bias, leading to false-negative results for certain binding sites.

#### DNA/protein-centered strategies

ChRD-PET uses antibodies against histone modifications to anchor specific genomic regions, captures RNA molecules that physically bind to them, and then performs in situ proximity ligation using a biotin-labeled DNA bridge linker [14]. This technology has been used to analyze caRNA interaction networks in H3K4me3-marked active promoter regions of rice, confirming that caRNAs are extensively involved in the formation of chromatin loops in plants.

TaDRIM-seq uses Protein G-Tn5 transposase to target specific chromatin regions; after inserting sequencing adapters, it simultaneously captures DNA-RNA and RNA-RNA interactions through in situ proximity ligation [15]. This technology has been validated in animal CTCF sites and plant circadian responses and is currently the only targeted technology capable of simultaneously capturing both DNA-RNA and RNA-RNA interactions.

ChRD-PET uses histone modifications as anchors and is suitable for elucidating the composition of caRNAs in specific chromatin states; however, it cannot capture caRNAs that interact with unmarked chromatin regions. TaDRIM-seq uses DNA-binding proteins or specific DNA elements as anchors and offers higher resolution; however, it relies on the targeting efficiency of Protein G-Tn5, and the compatibility of different antibodies still requires systematic evaluation.

### Non-targeted identification techniques

Non-targeted caRNA identification technologies are methods that do not require the pre-selection of any specific RNA or DNA targets, but instead enable the unbiased, one-time capture of physical interactions between all RNA molecules and all DNA sites across the entire genome. These primarily include GRID-seq (Global RNA Interactions with DNA by deep sequencing) [21], iMARGI (in situ Mapping of RNA-Genome Interactions) [22], RADICL-seq (RNA and DNA Interacting Complexes Ligated and sequenced) [23], ChAR-seq (Chromatin-Associated RNA sequencing) [24], and Red-C (RNA ends on DNA Capture) [25].

#### DNA/protein-centered strategies

As a pioneer in non-targeted caRNA capture technology, the GRID-seq method employs a bifunctional linker, with one end attached to double-stranded DNA and the other to single-stranded RNA, to covalently link spatially adjacent RNA and DNA molecules following restriction enzyme digestion of chromatin [21]. This technology has, for the first time, enabled the comprehensive capture of caRNA interactions and can effectively distinguish between cis and trans interactions. Its application in *Arabidopsis thaliana* revealed that mRNAs primarily mediate cis-local interactions, while lncRNAs dominate long-range interchromosomal interactions [26]. Studies in porcine skeletal muscle revealed that caRNAs encoding troponin T (TTN) are widely distributed across the genome and may be extensively involved in the regulation of skeletal muscle function through a trans-acting mechanism [27].

iMARGI shifts the proximity-linking reaction from in vitro magnetic beads to in situ within intact cell nuclei, thereby reducing structural changes caused by in vitro manipulation [22]. By introducing RNase A pretreatment to selectively remove free RNA, iMARGI can enrich stable, protein-protected RNA-chromatin interactions. It has been found that both short-range and long-range interactions are resistant to RNase and structurally stable, and the method highly enriches disease-associated nuclear lncRNAs [28].

RADICL-seq employs the RNase H enzyme to specifically digest RNA-DNA hybrids in transcriptionally active regions, thereby reducing background noise caused by novel transcripts and rRNA [23]. This technique has provided a detailed map of the genome-wide interaction patterns of the lncRNA Neat1 in mouse cells, confirming that its long isoforms alter the three-dimensional conformation of chromatin through trans-interactions.

ChAR-seq uses a 5′-adenylated single-stranded DNA bridge linker to connect the 3′ end of RNA via a mutant T4 Rnl2tr enzyme, which is then linked in situ to fragmented chromatin DNA to form asymmetrically polarized RNA-oligo-DNA chimeric fragments [24]. This design allows for the differentiation between the RNA-derived and DNA-derived ends during sequencing. In Drosophila S2 cells, it was found to not only capture a large number of co-transcribed nascent RNAs and trans-regulatory elements but also precisely capture the interaction network of the dose-compensating lncRNA roX2 [24]. In the non-model species lizard, three lncRNAs with trans-regulatory features were identified that showed significant spatial colocalization with H4K16ac modification signals. This finding suggests regulatory potential for lncRNA prediction in the absence of functional annotations [29].

All four technologies are based on the principle of proximity-based linking, but they differ in linker design, linking sites, and background control. The linker design of GRID-seq effectively distinguishes between cis and trans interactions, but its resolution is limited by the distribution of restriction enzyme cleavage sites. iMARGI’s in situ ligation strategy better preserves the natural structure within the nucleus, but it requires a higher starting cell count. RADICL-seq effectively reduces background from nascent RNA through RNase H treatment, but excessive digestion may lead to the loss of true R-loop signals. ChAR-seq has relatively low throughput, and its data analysis workflow is not yet as mature as that of GRID-seq. However, all four of these techniques are based on the principle of proximity linking, The RNA-DNA interactions it captures, at the molecular level, essentially span a continuum ranging from stable physical binding to spatial proximity [30]. For example, some of the signals detected by GRID-seq and iMARGI may reflect spatial colocalization within transcriptional factories or nuclear compartments rather than sequence-specific functional interactions [31]. Therefore, caRNA-chromatin associations identified by non-targeted techniques require functional validation through targeted techniques or genetic perturbation experiments.

#### Global capture and targeted protein strategies

Red-C digests chromatin with the DpnII restriction endonuclease and introduces a specific bridge adapter to directly link the 3′ end of an RNA molecule to fragmented DNA, thereby capturing information from both ends of the RNA and its interacting DNA sites. Research has shown that in the human K562 cell line, miRNAs tend to interact with heterochromatic regions, while eRNAs preferentially engage in cis-interactions with other enhancers on the same chromosome [25].

Subsequently, the researchers combined Red-C with chromatin immunoprecipitation (ChIP) to develop the RedChIP technique, which specifically targeted the EZH2 core subunit and the insulator protein CTCF, thereby mapping a non-coding RNA landscape associated with multi-comb repressive elements and topological boundaries [32]. BRG1-RedChIP further targeted the SWI/SNF catalytic subunits and revealed that a large number of trans-acting lncRNAs were co-enriched with the SWI/SNF complex in cell-type-specific enhancer regions [33].

The RedChIP series of techniques resolves RNA-chromatin interactions down to specific protein-mediated subclasses, but its coverage is narrower than that of the global Red-C method [33], making it more suitable for the detailed analysis of specific protein complexes. Furthermore, because this technique incorporates a ChIP step, data quality may be affected by antibody specificity and immunoprecipitation efficiency. The major whole-genome DNA–RNA interaction mapping technologies, together with their principles, technical biases, detectable interaction types, and representative applications, are summarized in Table 1.

**Table 1.** Comparison of techniques for identifying RNA-Chromatin interactions.

Technology Name	Targeted/Non-targeted	Anchor Types	Technical Principles	Major Technical Biases	Detectable Interaction Types	Notable Discoveries	References
ChIRP-seq	Targeted	RNA-centric	Biotinylated oligonucleotide probes	Probe accessibility, off-target hybridization	Protein-mediated bridging, spatial proximity	*HOTAIR* colocalizes with PRC2	[16]
CHART-seq	Targeted	RNA-centric	Complementary probes for RNase H	Secondary structure dependence	Protein-mediated bridging, spatial proximity	*Xist* dynamically covers the X chromosome	[17]
ChRD-PET	Targeted	Protein/DNA-centric	Combining ChIP with an in situ proximity ligation strategy	Antibody specificity bias	Protein-mediated bridging	Rice caRNAs participate in chromatin loops	[14]
TaDRIM-seq	Targeted	Protein/DNA-centric	Targeted probes combined with in situ proximity ligation	Tn5 targeting efficiency	Direct contact, protein bridging, RNA-RNA	Circadian rhythm of the rice caRNA network	[15]
GRID-seq	Non-targeted	–	Specific bifunctional Linker	Restriction enzyme sites, rRNA background	Spatial proximity, direct contact	Arabidopsis lncRNAs dominate trans-interactions	[26]
iMARGI	Non-targeted	–	RNA terminal modifications are directly linked to DNA	Ligation efficiency	Spatial proximity, stable protein-protected interactions	mt-caRNAs anchor active promoters	[28]
RADICL-seq	Non-targeted	–	Treatment with RNase H in conjunction with bridged oligonucleotides	Excessive RNase H digestion	Spatial proximity	*Neat1* trans-modulates chromatin conformation	[23]
ChAR-seq	Non-targeted	–	In situ ligation of oligonucleotide adapters	Ligase efficiency	Spatial proximity, direct contact	Lizard lncRNAs colocalize with H4K16ac	[29]
Red-C	Non-targeted	–	Optimized bridging and multiple restriction enzyme digestion	Restriction enzyme sites	Spatial proximity, direct contact	eRNAs prefer cis-acting enhancer interactions	[25]
RedChIP	Targeted	Protein-centric	ChIP+Red-C	ChIP efficiency	Specific protein-mediated bridging	Co-enrichment of lncRNA and SWI/SNF	[33]

## A comparative analysis of the mechanisms underlying functional conservation and specificity in plants and animals

As a central regulatory factor in the chromatin microenvironment, caRNA plays a crucial role in transcriptional activation, silencing, and the maintenance of three-dimensional chromatin structure through various mechanisms, including cis-anchoring, trans-recruitment, and R-loops. Although these mechanisms are widespread in eukaryotes, the specific molecular mechanisms and regulatory patterns differ between animals and plants, exhibiting both conservation and divergence. This subsection explores the functions of caRNA across four dimensions: the role of caRNA in organizing three-dimensional chromatin topology; m^6^A modification as a conserved chromatin regulatory switch; caRNA-mediated environmental and developmental signaling; and the pathological mechanisms mediated by caRNA network dysregulation.

### caRNA as an organizer of three-dimensional chromatin topology

#### Global and local topological regulation of chromosomes in animals

In animal systems, studies have shown that caRNAs play a crucial role as key components of three-dimensional chromatin architecture. The long non-coding RNA (lncRNA) Xist, a specific transcript of the inactivated mammalian X chromosome, is a classic example. Xist is anchored cis-wise to its transcriptional source, the inactivated X chromosome (Xi), in a manner dependent on three-dimensional chromatin topology; it then interacts over long distances along the entire chromosome. Due to the three-dimensional folding of chromatin, it in situ recruits the trans-diffusing PRC2 complex and catalyzes the repressive H3K27me3 modification, ultimately achieving transcriptional silencing of the entire X chromosome [34,35].

In addition to Xist-mediated whole-chromosome silencing, caRNA also participates in the local topological regulation within TADs and in enhancer-promoter loops. Take the Cpox gene, located at the boundary of a TAD, as an example: its pre-mRNA is not processed like a typical mRNA precursor but remains on the chromatin, linking the gene body region to an adjacent enhancer via cis-action to form a repressive loop structure. When this pre-mRNA is knocked down, PRC2 dissociates from the region, leading to a subsequent reduction in H3K27me3 modifications at the Cpox locus; p300 is released, and multiple target genes within the TAD are abnormally activated [36]. In other words, the anchoring of pre-mRNA to chromatin can alter the local three-dimensional structure, allowing it to exert its function even before it is translated into protein.

During tumorigenesis, the colorectal cancer-specific lncRNA CCAT1-L accumulates in the cell nucleus after transcription rather than being transported to the cytoplasm, where it acts as a cis-anchoring molecule in specific super-enhancer regions. CCAT1-L, retained in the nucleus, binds to CTCF to promote and maintain, in situ, long-range chromatin looping between the MYC oncogene promoter and a distal enhancer, thereby altering the local three-dimensional chromatin structure and contributing to enhanced oncogenic transcriptional output. Targeted intervention to remove this transcript in situ disrupts the aforementioned oncogenic topological structure and reduces MYC expression^45^. This mechanism confirms a direct link between the spatial localization of caRNA and disease-associated chromatin remodeling.

#### Global and local topological regulation of chromosomes in plants

In plant models, the most distinctive and best-studied mechanism of caRNA in chromatin topology is the RNA-directed DNA methylation (RdDM) pathway. In this pathway, Pol V transcribes long non-coding scaffold RNAs, which persistently bind to chromatin and serve as molecular anchor points for the AGO4-siRNA complex. The AGO4 protein, which carries siRNAs, forms base pairs with the Pol V transcripts, thereby recruiting the de novo DNA methyltransferase DRM2 to catalyze DNA methylation at target sites [37–39]. This mechanism transforms Pol V transcripts into functional caRNAs that organize local heterochromatic structures by recruiting silencing complexes in a reciprocal manner. The molecular relationship among RdDM-derived small RNAs, Pol V-derived scaffold RNAs functioning as caRNAs, AGO4-mediated chromatin targeting, and DRM2-dependent DNA methylation, together with the major biological functions of the RdDM pathway, is summarized in Figure 2.

**Figure 2.**
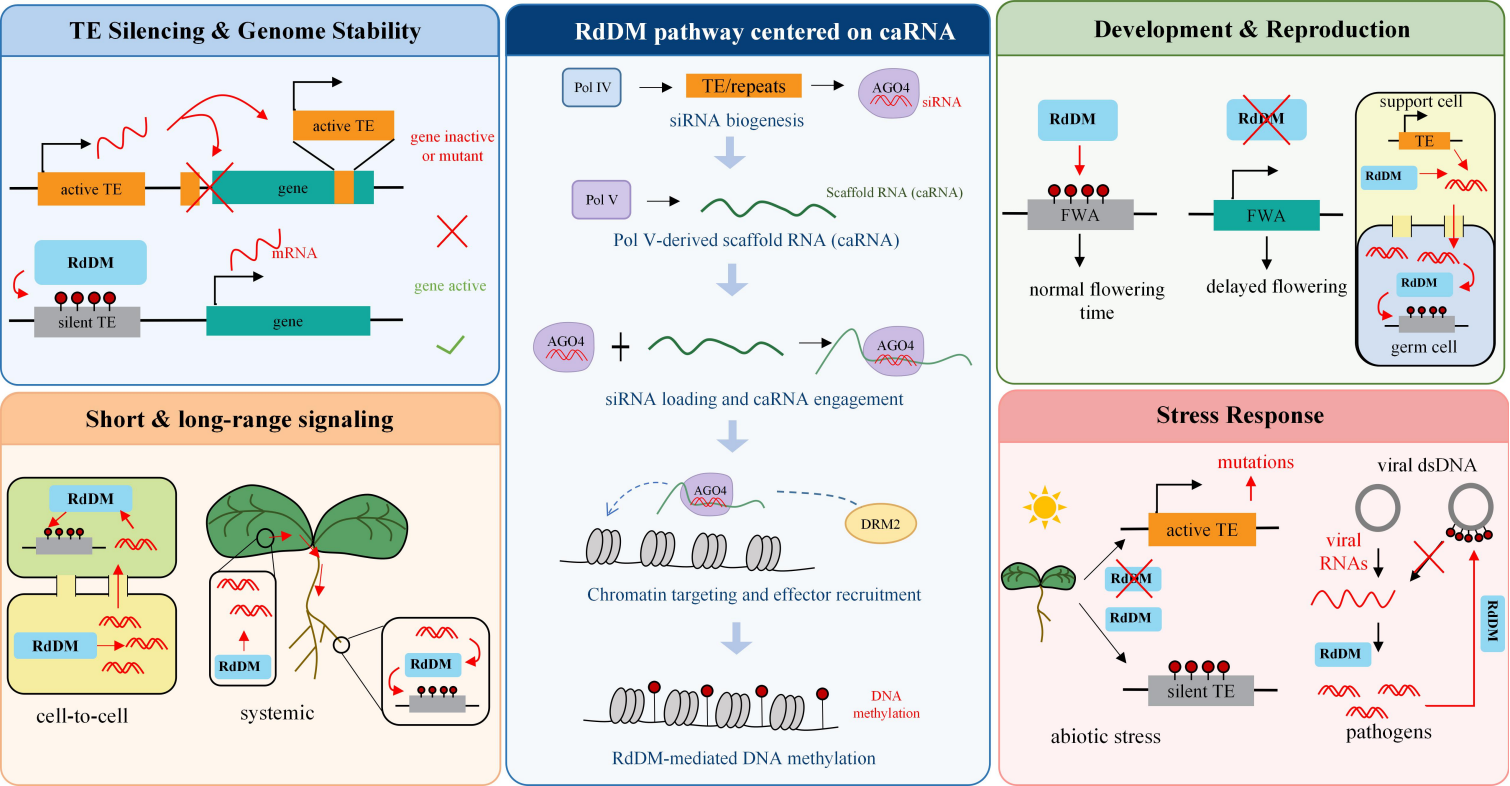
caRNA-centered RdDM pathway and its biological functions in plants.

The central panel illustrates the molecular mechanism of RNA-directed DNA methylation (RdDM) centered on chromatin-associated scaffold RNA (caRNA). Pol IV-dependent transcription of transposable elements (TEs) and repetitive sequences contributes to the production of 24-nt small interfering RNAs (siRNAs), which are loaded into AGO4. In parallel, Pol Vproduces scaffold RNAs that function as caRNAs at RdDM target loci. The AGO4–siRNA complex associates with Pol V-derived scaffold RNA through sequence-dependent RNA–RNA base pairing, facilitating chromatin targeting and recruitment of the denovo DNA methyltransferase DRM2. DRM2 subsequently catalyzes DNA methylation at targeted loci, leading to transcriptional silencing of TEs and regulation of gene activity. The surrounding panels summarize major biological functions associated with RdDM, including TE silencing and genome stability, developmental and reproductive regulation, short- and long-range signaling, and responses to abiotic and biotic stresses.

A systematic characterization of caRNA-chromatin interactions provides genome-wide evidence for the involvement of caRNAs in the three-dimensional genomic structure of plants. Xiao et al. [14] used the ChRD-PET technique to construct a high-resolution map of H3K4me3 and H3-associated RNA-DNA interactions in rice and demonstrated that caRNAs are extensively involved in the formation of chromatin loops and spatial chromatin interaction domains. Recently, Ding et al. [15] used TaDRIM-seq technology to target CTCF-marked chromatin loop anchor regions and successfully identified a widely enriched caRNA network. This map not only reveals interactions between classic lncRNAs such as MALAT1 and NEAT1 and CTCF-binding sites but also precisely captures the RNA-RNA interaction between NEAT1 and MALAT1. It also demonstrates that the caRNA interaction network in rice undergoes significant changes with the photoperiod. For example, the core circadian gene OsLHY forms a highly dense RNA-RNA interaction network only in the morning, whereas the RNA-DNA spatial interaction network appears dispersed in the evening.

#### Conservatism and specificity of caRNA in three-dimensional chromatin regulation in plants and animals

Both animals and plants utilize caRNA as a scaffold for three-dimensional chromatin structure, and their core mechanisms share commonalities, functioning through cis-anchoring or trans-recruitment. However, there are notable differences between the two in their reported modes of action. In animals, evidence for caRNA involvement in chromatin topological regulation spans multiple levels, ranging from the entire X chromosome to within TADs and enhancer-promoter loops. In contrast, the caRNA-mediated topological regulation currently well-established in plants is primarily confined to localized chromatin loops, such as the scaffold RNAs in the RdDM pathway and the chromatin loops captured by ChRD-PET. It remains unclear whether caRNAs similar to Xist, capable of mediating regulation at the whole-chromosome scale, exist in plants.

This difference in the scale of action may stem from the distinct biological functions that caRNAs perform in animals and plants. In plants, the primary role of the RdDM pathway is to establish DNA methylation at transposon sites; it does so by modifying each site independently rather than covering the entire chromosome. Pol V transcribes a scaffold RNA at each target site, recruits AGO4 and DRM2 to complete the local modification, and then dissociates. In contrast, the role of Xist in animals is to completely silence an entire X chromosome, causing caRNA to spread along the entire chromosome from a single starting point. Therefore, the absence of Xist-like caRNA in plants does not indicate that plants lack the potential to implement this mechanism; rather, the functional requirements of the RdDM pathway itself do not involve chromosome-wide coverage, a point that warrants further investigation.

### m^6^A modification as a conservative switch for chromatin regulation

For a long time, m^6^A has been widely recognized as a key chemical modification that regulates the translation of mature mRNAs. m^6^A modifications are also highly enriched on caRNAs and play an important role in epigenetic regulation. In animals, the regulatory role of m^6^A on caRNA has been validated across various RNA substrates, including Xist, LINE1, and eRNA; in plants, related studies have focused on the COOLAIR pathway and transposon silencing pathways in Arabidopsis. Research in both domains collectively demonstrates that m^6^A modification serves as a functional switch for caRNA in both animals and plants, although its downstream mechanisms of action exhibit marked differences between the two kingdoms.

#### Nuclear m^6^A recognition and chromatin regulation in animals

N^6^-methyladenosine (m^6^A) is widely recognized as a key chemical modification that regulates the translation of mature mRNAs, influencing both the translation and stability of cytoplasmic mRNAs. A growing body of evidence indicates that m^6^A modifications are also highly enriched on chromatin-associated RNAs and play a crucial role in epigenetic regulation. In a landmark study, Liu et al. [40] demonstrated that METTL3 catalyzes the transcriptional co-deposition of m^6^A on chromatin-associated regulatory RNAs (carRNAs), and that the nuclear m^6^A reader protein YTHDC1 recognizes this modification and specifically promotes the nuclear degradation of carRNAs; knockout of Mettl3 or Ythdc1 leads to abnormal accumulation of carRNAs, resulting in abnormal chromatin opening. In contrast, the cytoplasmic m^6^A reader protein YTHDF2 primarily promotes mRNA translation suppression and degradation in the cytoplasm by recruiting the CCR4-NOT complex; the two proteins exhibit fundamental differences in their subcellular localization and functional mechanisms.

m^6^A-modified caRNA can act as a molecular scaffold to mediate heterochromatin formation and transcriptional silencing. YTHDC1 promotes X-chromosome transcriptional repression by recognizing and binding to m^6^A on the lncRNA XIST [41]. A similar mechanism is observed in mouse embryonic stem cells, where YTHDC1 promotes the formation of the LINE1-NCL protein complex by recognizing m^6^A modifications on LINE1 RNA. This complex subsequently recruits the KAP1/TRIM28 and SETDB1 complexes, establishing the repressive H3K9me3 mark, thereby maintaining stem cell pluripotency and genomic stability [42,43]. Furthermore, the demethylase FTO directly removes m^6^A modifications from LINE1 RNA; this removal process directly regulates the abundance of LINE1 RNA on chromatin, thereby controlling local chromatin states in mouse oocytes and early embryonic development [44].

The m^6^A-modified caRNA network also plays a role in maintaining the three-dimensional chromatin conformation of the genome. In an acute myeloid leukemia (AML) model, the METTL3-YTHDC1 axis mediates m^6^A modification of structural long noncoding RNAs (lncRNAs), a process that is critical for regulating CTCF binding and maintaining the integrity of transcription activity domain (TAD) boundaries [45]. Furthermore, m^6^A modifications on enhancer RNAs (eRNAs) serve as activation signals. m^6^A modification of enhancer RNAs (eRNAs) promotes enhancer and adjacent gene activation by recruiting the nuclear m^6^A reader YTHDC1, which facilitates the formation of BRD4-associated transcriptional condensates [36]. m^6^A not only modifies single-stranded RNA but is also highly enriched on RNA strands within R-loops. Intranuclear m^6^A recruits YTHDC1, which specifically recognizes these R-loops and, by recruiting helicases, prevents DNA double-strand breaks, thereby forming a critical link between transcription and genomic stability [46].

#### Nuclear m^6^A recognition and chromatin regulation in plants

The m^6^A modification system in plants is both functionally conserved and distinctly different from that in animals. Although plants lack homologs of the YTHDC1 gene found in mammals, they have evolved a unique family of ECT proteins, which primarily regulate post-transcriptional gene expression in the cytoplasm [47]. However, at the level of nuclear chromatin, m^6^A modifications on caRNAs also serve as a key hub for epigenetic regulation in plants.

A striking example is the COOLAIR-FCA-FLD pathway in Arabidopsis. The long non-coding RNA COOLAIR forms an R-loop at the FLC locus upon cold induction. The RNA-binding protein FCA, together with its cofactor FLL2, promotes m^6^A modification on COOLAIR. The m^6^A modification enhances FCA binding and facilitates FLL2-dependent aggregate formation. These aggregates recruit 3′-truncated R-loops and are coupled with FLD-mediated H3K4me1 demethylation, ultimately leading to in situ chromatin silencing of the target gene FLC [48,49]. This mechanism links m^6^A modification, phase separation, and chromatin silencing, forming a plant-specific molecular pathway. Recently, Song et al. [50] systematically mapped the m^6^A methylation profile of Arabidopsis transposon RNAs and revealed that the FIP37 and VIR subunits first add m^6^A modifications to newly synthesized transposon caRNAs. CPSF30-L specifically recognizes this modification and preferentially recruits the histone methyltransferases SUVH4/5/6 and ATXR5/6, which establish H3K9me2 and H3K27me1 marks at target sites, thereby converting active transcriptional signals into heterochromatin silencing. This pathway provides a plant-specific example of m^6^A-dependent heterochromatin formation.

#### Conservatism and specificity of m^6^A modification in chromatin regulation in plants and animals

The use of m^6^A as a regulatory switch for caRNA is a highly conserved functional principle in both the animal and plant kingdoms. In animal systems, the nuclear m^6^A reader protein YTHDC1 recognizes m^6^A modifications on caRNA, mediates its degradation, and thereby regulates local chromatin states; whereas in plants, m^6^A recruits histone methyltransferases via the FIP37–CPSF30–SUVH axis to catalyze the establishment of repressive histone marks such as H3K9me2, thereby achieving transcriptional silencing of chromatin. Notably, although the core function of m^6^A modification is highly conserved across species, its downstream molecular reading and effector mechanisms exhibit significant phylogenetic specificity. Animals primarily rely on YTHDC1 as the nuclear m^6^A recognition and effector platform, whereas plants have independently evolved a unique reading protein system represented by CPSF30-L and ECT family proteins. Taken together, these findings support the notion that m^6^A-mediated chromatin regulation follows a pattern of convergent evolution, wherein animals and plants independently evolved their own specific reading protein and effector protein modules during the course of evolution, both of which implement the core molecular mechanism of regulating chromatin state through m^6^A modification.

Functional evidence across species further confirms the conserved role of m^6^A in chromatin regulation. When the human-derived m^6^A demethylase FTO is heterogeneously expressed in rice, exogenous FTO can efficiently and specifically remove m^6^A modifications from endogenous transcripts in rice. This cross-species demethylation led to a dramatic decrease in the levels of H3K9me2 and H3K27me3 modifications in rice, thereby triggering the activation of transcriptional networks by increasing chromatin accessibility [51]. These results demonstrate that the m^6^A modification of caRNA dynamically regulates chromatin state and serves as a core epigenetic mechanism in the evolution of plants and animals.

### The role of caRNA in the environment and transgenerational inheritance

As dynamic regulators of the chromatin microenvironment, caRNAs not only participate in the organization of structural chromatin but also serve as mediators for the perception and transmission of environmental information. In animals, it has been demonstrated that the caRNA network retained in sperm can carry paternal environmental memories; in plants, nuclear caRNAs integrate photoperiod, temperature, and stress signals in real time to regulate developmental processes. This section first describes the transgenerational epigenetic mechanisms mediated by caRNA in animal sperm, then analyzes the real-time regulatory functions of plant caRNA in circadian rhythms and stress responses, and finally integrates and compares the two from the perspectives of temporal scales and information transmission strategies.

#### DRNA in animal sperm and transgenerational epigenetics

Sperm caRNA Networks and Transgenerational Epigenetic Transmission In traditional reproductive biology, sperm serve as carriers of paternal DNA. However, the complex caRNA network within sperm and its physical spatial association with chromatin have been shown to act as vehicles for transmitting environmental memory. During the extreme processes of spermatogenesis and nuclear condensation, some RNAs are not completely cleared but are retained as components of chromatin. Using cell fractionation techniques, researchers identified a unique class of ‘DNA-associated RNA (DRNA)’ in mature mouse sperm. This DRNA is highly sensitive to RNase H and is tightly anchored to the paternal genome in the form of R-loops [52]. Whole-genome R-loop sequencing via DRIPc-seq confirmed that caRNA is a component of sperm chromatin, and that R-loops are not only distributed in telomeric regions but are also widely present in non-telomeric repetitive sequences and sex chromosome-specific loci, such as Erdr1 on the mouse Y chromosome and Asmt on the X chromosome [53].

Mature sperm nuclei retain a variety of functional RNAs, including nuclear-localized spliceosome snRNAs (U1/U2), small RNAs that establish H3K9me3 repressive marks in transposon regions via the piRNA pathway [54], and Ago4-associated small RNAs that localize to sex chromosomes and regulate meiosis-specific sex chromosome inactivation (MSCI) [55]. Furthermore, sperm-derived tRNAs and miRNAs can interfere with the degradation or expression of key target genes during zygotic genome activation (ZGA), thereby regulating the transcriptional network of the early zygote [56].

Environmental factors faced by the parental generation – such as disease, drug exposure, or psychological stress can directly reshape the caRNA expression profile of sperm. In a study using an endocrine disruptor exposure model, altered ncRNAs in F1-generation sperm showed a high frequency of spatial colocalization with DNA methylation differences (DMRs) and histone retention regions (DHRs) in the genomes of F1- to F3-generation offspring [57]. When sperm carrying this complex epigenetic information enter the egg cell, structural caRNAs attached to the sperm nuclear matrix maintain specific spatial conformations of paternal chromatin. After fertilization, it is hypothesized that the nucleosome-bound DNA retained in the sperm may mark nuclear matrix attachment regions (S/MARs), which anchor decompressed DNA loops and participate in the reconstruction of paternal nuclear architecture [58]. These observations suggest that specific caRNA structures can bypass early epigenetic reprogramming in the zygote, transmit epigenetic signals to the oocyte, and mediate the transgenerational inheritance of paternal traits [59–61].

caRNA Networks Governing Circadian Rhythms and Stress Responses in Plants

Since plants cannot move to avoid adverse environmental conditions, they have evolved chromatin regulatory networks to respond to environmental changes. In model plants such as Arabidopsis and rice, lncRNAs within caRNA compartments have been shown to be key mediators of environmental responses. Ding et al. [15] used the TaDRIM-seq technique to discover that within the chromatin loop-anchored regions of rice, there exists a caRNA interaction network that varies significantly with the photoperiod. For example, the core circadian gene OsLHY forms a highly dense RNA-RNA interaction network only in the morning, whereas the RNA-DNA spatial interaction network in the evening appears dispersed. This periodic oscillation of chromatin structure enables plants to anticipate and adapt to daily environmental fluctuations. At the molecular level, epigenetic enzymes act on chromatin during circadian oscillations, thereby maintaining the dynamic rhythmic expression of clock genes [62,63].

At the molecular level, epigenetic enzymes and lncRNAs synergistically regulate circadian chromatin states. The core circadian transcription factors CCA1 and LHY directly suppress the expression of the H3K4me3 demethylase JMJ14 while simultaneously activating the H3K4me3 methyltransferase SDG2, thereby driving circadian oscillations in histone modifications at target sites [63]. During the evening, ELF3 – a core component of the Evening Complex – specifically recruits the demethylase JMJ29 to the promoter regions of CCA1 and PRR9, maintaining their low expression levels by catalyzing H3K4me3 demethylation [64].

In addition to histone modifiers, lncRNAs also directly regulate plant circadian rhythms. FLORE, an endogenous antisense transcript in Arabidopsis, is regulated by the circadian clock, and its expression pattern is opposite to that of its sense gene, *CDF5*. FLORE suppresses multiple CDF family members through a trans-acting mechanism, thereby upregulating the transcription of FT genes. The CDF5-FLORE regulatory module integrates circadian and photoperiod signals, enabling plants to adjust their flowering time in response to environmental conditions [65].

#### A comparison of caRNA-Mediated transgenerational inheritance and environmental responses in plants and animals

Both animals and plants use caRNA as a carrier of environmental information, but there are significant differences between the two in terms of the temporal scale and direction of information transmission. Animals achieve transgenerational transmission via DRNA in sperm; environmental information experienced by the father is stored in sperm in the form of caRNA structures, which can partially evade early epigenetic reprogramming of the fertilized egg, thereby influencing the phenotype of the offspring. In addition to DRNA, sperm tsRNA and miRNA have also been shown to participate in the transgenerational transmission of metabolic diseases and stress responses [66,67]. In nematodes and fruit flies, the piRNA/22 G-RNA system can even maintain transposon silencing as a transgenerational epigenetic memory for hundreds of generations [68,69].

In contrast, caRNAs in plants can rapidly respond to circadian rhythms and seasonal changes, integrating environmental signals across temporal scales to drive the plasticity of developmental programs. For example, the RNA-RNA interaction network of the core circadian gene OsLHY undergoes significant reorganization in response to the photoperiod [15]. In Arabidopsis responding to pathogen infection, the nuclear-enriched lncRNA ELENA1 enhances the transcriptional activation of the defense gene PR1 by interacting with the Mediator complex [70]. Under drought stress in Arabidopsis and rice, m^6^A levels on the transcripts of stress-responsive genes such as RD29B and KIN1, and other stress-responsive genes [71]. Animals achieve a form of predictive adaptation through transgenerational transmission, whereas plants rely more on continuous responses to current environmental conditions. Notably, small RNA- and DNA methylation-mediated transgenerational inheritance has been confirmed in plants [72], but its molecular carriers differ fundamentally from DRNA in animal sperm. Plants primarily rely on mobile siRNAs and the RdDM pathway to maintain transposon silencing, rather than stable RNA structures anchored to chromatin in the form of R-loops. Therefore, it remains unclear whether plants possess a transgenerational anchoring mechanism for chromatin-bound RNAs similar to sperm DRNA.

### caRNA network dysfunction and disease onset

The maintenance of the steady state of the caRNA interaction network is a prerequisite for chromatin function; its disruption leads to dysregulation of gene expression and genomic instability. Due to biological differences between animals and plants, the manifestations of caRNA network disruption in these two kingdoms are markedly different. This section first systematically summarizes the types of molecular mechanisms by which caRNA network abnormalities promote tumorigenesis in animals, then describes the patterns of caRNA network reprogramming and genomic responses in plants under stress conditions, and finally provides a comparative analysis of caRNA functional dysregulation across species.

#### Abnormal caRNA regulation and tumor pathogenesis in animals

Specific disruptions in caRNA networks have been observed across various types of cancer and can be categorized into the following four types based on their mechanisms of action.

Disruption of the enhancer-promoter topological structure. Taking the lncRNA CCAT1-L, which is specifically overexpressed in colorectal cancer, as an example. This RNA remains in the nucleus after transcription and, by binding to CTCF, promotes long-range chromatin looping between the MYC oncogene promoter and a distal enhancer. Targeted removal of CCAT1-L disrupts this loop structure and downregulates MYC expression [73]. This finding suggests that cancer cells can exploit the nuclear localization and chromatin looping capabilities of caRNAs to maintain high levels of oncogene expression.

R-loop accumulation and genomic instability. Mutations in the tumor suppressor gene BRCA1 lead to the formation of pathological R-loops in the gene and enhancer regions of luminal cells, physically blocking the interaction between enhancers and promoters, which is associated with inhibited cell differentiation and the development of basal-like breast cancer [74]. In acute myeloid leukemia (AML), the abnormally overexpressed lncRNA HOTTIP anchors to CTCF-binding sites and forms R-loops; by stabilizing the CTCF/Cohesin complex, it reinforces the TAD boundaries of Wnt/β-catenin target loci, which is associated with abnormal transcription of oncogenes [75]. DDX41 mutations also lead to abnormal R-loop accumulation, triggering genomic instability and inflammatory responses, suggesting that they may accelerate AML progression [76]. Clinically, targeted drugs such as BET inhibitors and HDAC inhibitors have been shown to be associated with R-loop accumulation; regulating R-loop homeostasis may enhance the sensitivity of cancer cells to DNA damage, providing new insights for targeted therapy [4].

Abnormal recruitment of chromatin modification complexes. ncRNAs regulate gene expression by interacting with chromatin modification complexes. For example, the lncRNA HOTAIR can abnormally bind to and recruit the PRC2 complex to specific loci; its core subunit, EZH2, catalyzes the formation of the repressive H3K27me3 modification, which has been found to be associated with the silencing of tumor suppressor genes [77].

Interference with DNA damage repair. Following the occurrence of chromatin double-strand breaks, ATP-dependent chromatin remodeling complexes (such as SWI/SNF, ISWI, and CHD) are recruited to the damage site, where they promote the accessibility of repair factors by remodeling the chromatin structure. Non-coding RNAs (such as DDRNA) transcribed at double-strand breaks participate in the coordination of the repair process through phase separation mechanisms [78]. caRNA-mediated defects in repair function are one of the major causes of tumor genomic instability.

#### Mechanisms of caRNA regulation in plants under abiotic and biotic stress

Compared with the extensive literature on caRNA network dysregulation in animal cancers, pathological studies on caRNA regulatory abnormalities in plants remain insufficient, primarily because plants lack proliferative disease models driven by somatic mutations that are analogous to malignant tumors in animals. Nevertheless, genetic evidence suggests that functional dysregulation of plant caRNA networks under stress conditions is closely associated with genomic instability and adaptive costs.

Under abiotic stress, the RdDM pathway dynamically reprograms DNA methylation at transposons and stress-responsive genes, thereby regulating plant adaptation [72,79]. For example, high temperatures can lead to inhibited Pol V activity and decreased siRNA levels, resulting in the transgenerational activation of transposons such as ONSEN [72]. Meanwhile, R-loop homeostasis is disrupted under oxidative stress; mutations in RNase H or helicases lead to abnormal R-loop accumulation, triggering DNA replication stalling and double-strand breaks. Furthermore, the m^6^A methylation profile undergoes global remodeling under drought stress, and MTA mutations result in dysregulation of stress-responsive gene expression and reduced drought tolerance [71].

Under biotic stress, small RNAs derived from viral genomes (vsiRNAs) are integrated into the RdDM pathway, forming an antiviral defense pathway involving caRNAs through chromatin silencing mediated by AGO4 and Pol V scaffold RNA [50,80]. Furthermore, during pathogen-induced responses in Arabidopsis, the nuclear lncRNA ELENA1 enhances chromatin accessibility of defense genes by interacting with the Mediator complex [70]. Research by Song et al. [50] further demonstrates that m^6^A modifications on transposon caRNAs and the subsequent establishment of H3K9me2 are critical for maintaining heterochromatin stability under stress conditions; Functional loss of FIP37 or CPSF30-L is directly associated with transposon reactivation and increased genomic instability [81], which in turn affects plant development and stress tolerance.

#### A comparison of caRNA network dysregulation in plants and animals

Compared to plants, pathological studies of caRNA networks in animals have been more extensively explored, primarily due to the well-established disease models and genetic manipulation systems in the field of cancer biology. In animals, the mechanisms by which caRNA dysregulation promotes tumorigenesis can be categorized into four types: disruption of topological structure, accumulation of R-loops, abnormal recruitment of chromatin modification complexes, and impaired DNA damage repair. Although plants lack pathological states that directly correspond to cancer, they similarly exhibit genomic instability and developmental abnormalities caused by caRNA network dysregulation under stress conditions. This difference reflects the fact that animals face the risk of cancer driven by somatic mutations, whereas plants face the challenge of maintaining genomic integrity under environmental stress. Nevertheless, animals and plants share a common core logic in caRNA function: the use of RNA as a dynamic scaffold to recruit chromatin modification complexes under specific spatiotemporal conditions. In animals, the focus is on developmental programming and disease-related processes, whereas in plants, the emphasis is on environmental sensing and stress adaptation. Therefore, plant caRNA research may draw upon the well-established functional validation strategies from animal cancer research to establish a more rigorous chain of causal evidence.

Representative caRNAs from plant and animal systems, together with their chromatin-targeting mechanisms, interaction modes, molecular interactions, epigenetic consequences, functional scales, and levels of causal validation, are summarized in Table 2. Taken together, these examples highlight both conserved and lineage-specific principles of caRNA-mediated chromatin regulation in plants and animals, including chromatin organization, recruitment of epigenetic regulators, and responses to developmental or environmental signals, as summarized in Figure 3.

**Figure 3.**
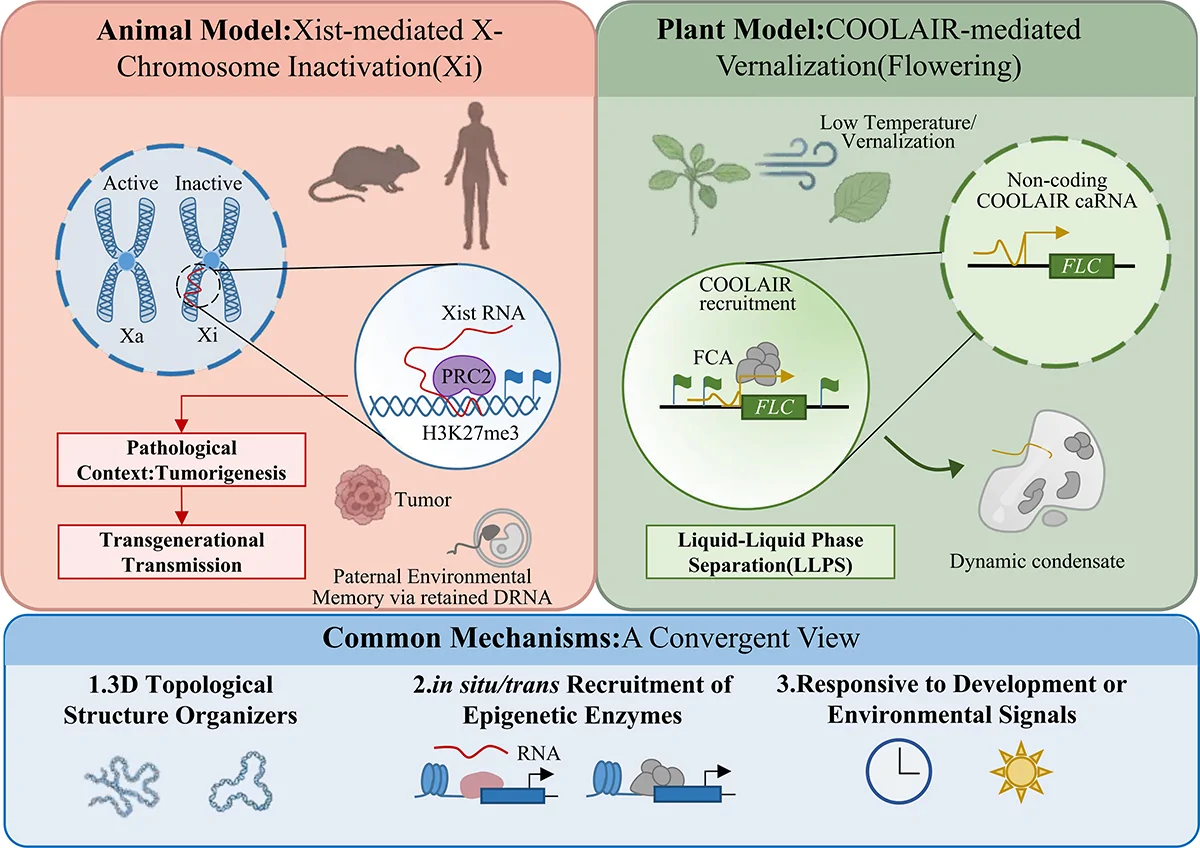
Comparative caRNA epigenetic mechanisms in animal and plant models.

**Table 2.** Representative caRnas and their mechanisms of chromatin targeting.

caRNA	Species/System	Core Mechanism Categories	Interaction Modes	Molecular Interactions	Epigenetic Consequences	Functional Scale	Level of Causal Validation
*Cpox* pre-mRNA	Mammals	3D Topology	cis-acting	Protein-mediated chromatin looping	H3K27me3 deposition	Local TAD	Knockdown Assays
Xist	Mammals	3D Topology	cis-trans cooperative	Protein Bridging (PRC2)	Chromosome-wide H3K27me3 deposition	Whole chromosome	Gene Knockout and Rescue Assays
CCAT1-L	Humans (Colorectal Cancer)	3D Topology	cis-acting	Protein Bridging (CTCF)	Enhancer-Promoter Cyclization	Long-range circularization	RNAi and CRISPR Assays
Pol V scaffold RNA	Plants (Arabidopsis/rice)	RNA-Guided Targeting	cis-acting	Protein Bridging (AGO4)	DNA Methylation	Local locus	Mutant and Rescue Assays
COOLAIR	Plant (Arabidopsis)	Modification-Dependent Regulation	cis-acting	R-loop+m^6^A Recognition	H3K4me1 Demethylation	Local locus	Mutant and Rescue Assays
LINE1 RNA	Mammals	Modification-Dependent Regulation	trans-acting	YTHDC1 Recognition	H3K9me3 Deposition	Repetitive sequence	Gene Knockout Assays
Transposon-derived caRNA	Plant (Arabidopsis)	Modification-Dependent Regulation	cis-acting	CPSF30-L Recognition	H3K9me2/H3K27me1 Deposition	Local locus	FTO Heterologous Expression Assays
DRNA	Mammals	Transgenerational Inheritance	cis-acting	R-loop Anchoring	DNA Methylation	Whole genome	Transplantation Experiments
FLORE	Plant (Arabidopsis)	Environmental Response Regulation	trans-acting	Protein Bridging	H3K4me3 activation-type modification	Multilocus	Mutant Assays

## Challenges and outlook

Although research on caRNA has made some progress in both plants and animals, the field still faces the dual challenges of widespread adoption of technology and translational applications on the path to a comprehensive understanding of non-coding RNA functions. Chromatin in the cell nucleus is highly folded and coiled, causing RNA to readily come into physical contact with open chromatin regions, which can easily lead to the generation of non-specific binding fragments. Furthermore, the vast majority of current RNA-chromatin interaction techniques rely on the mixed lysis of cell populations, resulting in heterogeneity among tissue cells at different developmental stages or throughout the developmental cycle. Physiological responses and tissue differentiation in organisms are, by their very nature, chromatin remodeling events occurring at the single-cell level. Therefore, the development of single-cell resolution caRNA capture technologies to systematically elucidate how specific loci remodel their local three-dimensional chromatin structure through the specific targeting of different caRNAs, thereby driving cellular differentiation into distinct lineages, will be a future trend and research direction. Single-cell caRNA capture technology helps systematically elucidate how specific loci are remodeled by the specific targeting of different caRNAs, reshaping the local three-dimensional chromatin structure and epigenetic regulatory landscape, and thereby driving cell specialization into different lineages.

Today, massive sequencing data face technical challenges such as high background noise and difficulty in controlling false positive rates. In the future, it will be necessary to integrate deep learning algorithms and large language models to predict key interaction nodes from multiple dimensions, including sequence features, three-dimensional structures, and evolutionary conservation [82]. Specific challenges in integrating artificial intelligence into the caRNA field include: distinguishing true interactions from experimental background noise in sequencing reads; predicting the secondary structure of caRNAs and its impact on probe accessibility to optimize ChIRP/CHART probe design; and integrating multi-omics data to construct interaction regulatory networks. However, the predictive results of AI models still require experimental validation before artificial intelligence can better support the elucidation of causal mechanisms in this field.

Looking ahead, as technologies such as AI-based data denoising models and single-cell spatial resolution continue to mature, the field of caRNA will not only deepen our understanding of basic life sciences but also provide new technical tools and directions for the development of novel anticancer drugs and the cultivation of future ‘super crops’ through epigenomic engineering. In the field of veterinary medicine, targeting caRNA-chromatin interaction networks offers new insights for the development of novel anticancer drugs. In the field of plant agriculture, breeding stress-tolerant crops by optimizing the RdDM pathway or m^6^A modification levels represents a key direction for translating basic research findings into agricultural applications.

## Data Availability

Data sharing is not applicable to this article as no new data were created or analysed in this study.
